# Partial weight support differentially affects corticomotor excitability across muscles of the upper limb

**DOI:** 10.14814/phy2.12183

**Published:** 2014-12-11

**Authors:** Keith D. Runnalls, Greg Anson, Steven L. Wolf, Winston D. Byblow

**Affiliations:** 1 Movement Neuroscience Laboratory, Department of Sport and Exercise Science, University of Auckland, Auckland, New Zealand; 2 Centre for Brain Research, University of Auckland, Auckland, New Zealand; 3 Department of Rehabilitation Medicine, Emory University School of Medicine, Atlanta, Georgia; 4 Atlanta VA Center of Excellence in Visual and Neurocognitive Rehabilitation, Atlanta, Georgia

**Keywords:** Gravity compensation, integrated control, intracortical inhibition, motor cortex, transcranial magnetic stimulation

## Abstract

Partial weight support may hold promise as a therapeutic adjuvant during rehabilitation after stroke by providing a permissive environment for reducing the expression of abnormal muscle synergies that cause upper limb impairment. We explored the neurophysiological effects of upper limb weight support in 13 healthy young adults by measuring motor‐evoked potentials (MEPs) from transcranial magnetic stimulation (TMS) of primary motor cortex and electromyography from *anterior deltoid* (AD), *biceps brachii* (BB), *extensor carpi radialis* (ECR), and *first dorsal interosseous* (FDI). Five levels of weight support, varying from none to full, were provided to the arm using a commercial device (Saebo Mobile Arm Support). For each level of support, stimulus–response (SR) curves were derived from MEPs across a range of TMS intensities. Weight support affected background EMG activity in each of the four muscles examined (*P *<**0.0001 for each muscle). Tonic background activity was primarily reduced in the AD. Weight support had a differential effect on the size of MEPs across muscles. After curve fitting, the SR plateau for ECR increased at the lowest support level (*P *=**0.004). For FDI, the SR plateau increased at the highest support level (*P *=**0.0003). These results indicate that weight support of the proximal upper limb modulates corticomotor excitability across the forearm and hand. The findings support a model of integrated control of the upper limb and may inform the use of weight support in clinical settings.

## Abstract (precis)

Partial weight support of the arm may hold promise as a therapeutic adjuvant during stroke rehabilitation, but little is known about its neurophysiological effects. We measured EMG activity and motor‐evoked potentials from TMS during variable weight support in healthy adults. Weight support had a differential effect on the size of MEPs across muscles, indicating support of the proximal upper limb modulates corticomotor excitability across the forearm and hand.

## Introduction

Functional linkages of muscles, or synergies, have been proposed as a biological mechanism for controlling complex motor systems (Kelso et al. 1980). For upper limb movements such as reaching, relative joint motions along the proximal–distal axis are strongly correlated (Lacquaniti and Soechting 1982; Soechting 1984). The neurophysiological basis of synergies linking distal and proximal muscles, and their physiological regulation remains an open question. For example, these may be embedded as circuits in the primary motor cortex (M1) (Park et al. 2001; Capaday et al. 2013). In humans, functional magnetic resonance imaging shows an overlap of muscle representations in M1 consistent with integrated control of upper limb muscles (Sanes et al. 1995). From TMS studies, the extent of activation of shoulder muscles can regulate healthy reach to grasp synergies by modulating corticomotor excitability of task relevant distal muscles (Devanne et al. 2002). If muscles involved in reaching movements are activated as a functional unit, it is possible that the organizational structure of the synergy places proximal muscles such as the *anterior deltoid* in a regulatory role within a hierarchy.

Understanding how synergies are organized and regulated is fundamental to developing better diagnostic tools and therapies for those with movement disorders or acquired deficits due to brain injury such as stroke. For stroke survivors, the central feature of the stereotyped flexor synergy pattern is an involuntary coupling of elbow flexor activity to antigravity torques at the shoulder (Dewald and Beer 2001). This loss of independent joint control restricts access to the normal workspace and compromises the ability to independently perform activities of daily living. At present, our understanding of the etiology and mechanisms of synergy expression in stroke survivors is limited. One promising approach may be to reduce shoulder torque requirements during reaching through partial weight support (Prange et al. 2009a). Partial weight support of the upper limb seems to reduce the deleterious effects of abnormal synergies, and permit patients a greater range of motion. However, the neurophysiological underpinning of these benefits is not yet known.

In healthy participants, corticomotor excitability (CME) to forearm muscles increases with anterior deltoid activation and this occurs at least in part, via disinhibition within the primary motor cortex (Devanne et al. 2002). Shoulder posture can also increase CME directed to distal hand muscles. For example, a horizontally adducted posture increases CME of hand and forearm extensors that serve to open the hand during grasping (Dominici et al. 2005). These findings are consistent with the hypothesis of a proximal–distal reaching synergy that is at least partly mediated within the primary motor cortex.

In this study, we sought to probe CME of descending motor pathways that comprise a putative upper limb synergy in healthy adults using transcranial magnetic stimulation (TMS). We expected that isometric contraction of *anterior deltoid* would modulate CME of forearm muscle, *extensor carpi radialis*. We investigated parametric weight support of the arm using a commercially available rehabilitation device that provided upward force to the arm through a forearm brace. To provide a reference, tonic background activity and motor evoked potentials were analyzed from muscles across the upper limb. We then examined CME and short‐latency intracortical inhibition (SICI) in forearm and hand muscles and hypothesized that an increase in support would lead to a decrease in CME distally. This was examined using TMS‐derived stimulus–response curves and paired‐pulse TMS for SICI as evidence in support of a cortical mechanism underlying upper limb synergy formation.

## Materials and Methods

### Participants

Thirteen right‐handed healthy young adults (six females) without history of upper limb injury or neurological illness participated in this study. The study was approved by the University of Auckland Human Participants Research Ethics Committee in accordance with the Declaration of Helsinki. Participants gave written informed consent and were screened for contraindications to TMS by a neurologist.

### Design

We utilized a single‐session repeated measures design in which all participants completed all task conditions. All muscles were examined simultaneously. Single‐pulse TMS was used to obtain stimulus–response curves at five levels of weight support. Paired‐pulse TMS was used to measure SICI at the minimum and maximum levels of weight support. The order of weight support was randomized between participants for stimulus–response curves and counterbalanced for SICI collection. Each session lasted approximately two hours.

### Posture and arm support

**Figure 1.**
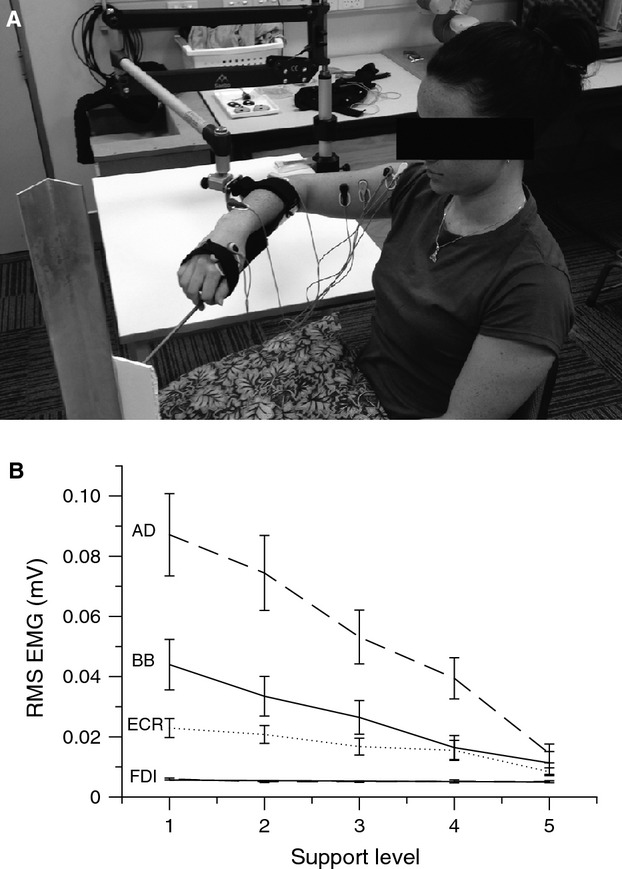
(A) Example of experimental arrangement. (B) Background muscle activity as a function of support level, ranging from minimal support (1) to full support (5). Average root‐mean‐square EMG of *anterior deltoid* (AD), *biceps brachii* (BB), *extensor carpi radialis* (ECR), and *first dorsal interosseous* (FDI) muscles from the prestimulus interval. Error bars represent ± SEM.

The experimental arrangement is illustrated in Fig. 1A. Participants were comfortably seated with their left arm resting on a cushion on their lap. The right arm was supported by a SaeboMAS dynamic mobile arm support system (Saebo Inc., Charlotte, NC). The SaeboMAS provided continuously adjustable weight support through a brace that cradled the proximal forearm. A rigid extension of the brace supported the wrist and hand. We utilized additional foam padding to support the forearm, wrist, and hand. All TMS was delivered in a standardized static posture that was voluntarily maintained by the participant. The shoulder was abducted 90° into the transverse plane and horizontally abducted 45° forward. The elbow was flexed to 90° and the forearm pronated. The SaeboMAS was set to prevent rotation of the brace in the vertical plane thus ensuring the forearm was always parallel to the floor. The wrist was neutral and the hand was relaxed. Joint angles were set using a goniometer. Participants would return to this set position by aligning a pointer that extended forward from the brace to a target.

The *Saebo*MAS permitted continuous manipulation of supportive force. We defined five equally spaced support levels from level 1, in which the device only compensated for its own weight and provided no significant support, to level 5, in which the device fully compensated for the weight of the arm. To determine the setting for level 5, we monitored activity in the *anterior deltoid* muscle in real time and incrementally decreased the supportive force from a high setting until root‐mean‐squared (rms) EMG was observed to deflect away from baseline.

### Electromyography and transcranial magnetic stimulation

Surface electromyography data were recorded from the right *anterior deltoid* (AD), *biceps brachii* (BB*), extensor carpi radialis* (ECR), and first *dorsal interosseous* (FDI) muscles. Following skin preparation, self‐adhesive 10‐mm‐diameter Ag‐AgCl electrodes (BlueSensor N; Ambu, Ballerup, Denmark) were arranged in a belly‐tendon montage for FDI and ECR, and a belly‐belly montage for BB and AD. A common ground electrode was placed over the acromion process (Red Dot: 3M Health Care, London, Canada). Signals were amplified (Grass P511AC; Grass Instrument Division, Warwick, RI) with 1000× gain, band‐pass filtered (3–1000 Hz), sampled at 2 kHz using a 16‐bit A/D acquisition system (National Instruments, Austin, TX), and saved to disk for subsequent offline analysis.

Single‐ and paired‐pulse TMS of left M1 was delivered using two MagStim 200 magnetic stimulators connected to a BiStim unit (Magstim, Dyfed, UK). A figure‐of‐eight coil (Magstim D70^2^) was held tangentially to the scalp and perpendicular to the central sulcus, with a posterior to anterior‐induced current flow. The coil was positioned at the site eliciting maximal motor‐evoked potentials (MEPs) in the resting right ECR muscle and the location was marked on the scalp. Consistent coil position and orientation were maintained through alignment of a template to the scalp markings prior to each stimulus. Resting motor threshold (RMT) was defined as the minimum stimulus intensity that elicited a 50 *μ*V MEP in four of eight trials. Active motor threshold (AMT) was defined as the minimum stimulus intensity that elicited a MEP in four of eight trials while maintaining wrist extension against gravity.

A stimulus–response curve was collected at each of the five arm support levels. A single stimulation site was used to concurrently elicit MEPs in all muscles. Ten stimulus intensities were set relative to RMT of ECR (−5, 0, +5, +10, +15, +20, +25, +30, +35, +40% of maximum stimulator output). For each curve, six stimuli were delivered at each intensity in a block‐randomized order. To mitigate fatigue, rest breaks were given following every three stimuli.

For paired‐pulse TMS the test stimulus (TS) intensity was set to produce nonconditioned (NC) MEPs of approximately half the observed maximum amplitude at rest. The conditioning stimulus (CS) intensity was set equivalent to AMT and delivered 2 ms preceding the TS. Sixteen stimuli (8 C&8 NC) were delivered in a randomized order at both the minimum and maximum levels of arm support.

### Data analysis

The average MEP area was used as the primary dependent measure. For each trial, baseline area was calculated as the integral of EMG over a 20‐ms interval immediately preceding stimulation. This was subtracted from the area integrated over a 20‐ms MEP window. MEP onset was determined manually for each muscle for each participant. As a secondary dependent measure, background EMG was calculated as the average pre‐trigger root‐mean‐squared EMG activity for each trial. To account for differences in MEP size between participants, MEPs were normalized relative to the largest mean MEP recorded in that muscle. Statistical analyses were then carried out using mean normalized MEP area and mean background EMG at each combination of stimulus intensity and support level. Mean normalized MEP areas were averaged across participants and the group level data were fit with three‐parameter sigmoidal Boltzmann functions using nonlinear regression (Devanne et al. 1997). The slope, s50, and plateau parameters collectively describe the recruitment properties of the pathway. While motor threshold is not explicitly represented, changes in CME of the most excitable neurons are captured by a shift in the s50 parameter. The regression procedure does not assume the plateau of the function exists within the range of sample data. For SICI, mean conditioned (C) and nonconditioned (NC) MEP areas were calculated and inhibition calculated as %SICI = 100 − (^C^∕_NC_ × 100). A change score for inhibition (ΔINH) was determined between minimum and maximum levels of support.

### Statistical analysis

Isometric muscle activity and MEP area were analyzed separately for each muscle using R 3.0.2 (R Core Team 2013) with the Linear and Nonlinear Mixed Effects Models package (Pinheiro et al. 2013). For isometric muscle activity, we performed a linear mixed‐effects analysis of the relationship between average root‐mean‐squared EMG and support level. We modeled *support level* and *stimulation intensity* as fixed effects with interaction terms, and *subjects* as a random intercept effect.

For normalized MEP area, data were logit transformed in order to meet the assumption of homoscedasticity. For normalized MEP area, a linear mixed‐effects analysis with *support level, stimulation intensity*, and *background* EMG activity as fixed effects and subjects modeled as a random intercept effect. To understand the effect of background EMG values on normalized MEP area for the ECR and FDI, pairwise comparisons were conducted on interpolated means from the statistical model at specified values of the background EMG covariate (Luo and Koolaard 2014).

Group stimulus–response curves for ECR and FDI were analyzed using extra sum‐of‐squares F‐tests in Prism (GraphPad, San Diego, CA) to assess whether individual curves for each support level fit the data significantly better than a single global curve for all support levels (Capaday et al. 1999). Differences in specific fit parameters of the group curves were analyzed using corresponding procedures to compare curves fit with independent parameters to those constrained to share the parameter of interest.

To examine SICI, conditioned MEP areas were analyzed using a linear mixed model. Two levels of *support* (*min, max*) and two levels of *stimulation (conditioned, nonconditioned)* were modeled as fixed effects with *background* EMG as a covariate. Subjects were modeled as a random intercept effect. In a second analysis, we tested whether SICI differed between the maximum and minimum support levels with a one‐sample t‐test to determine if ΔINH deviated from 0.

An alpha level of 0.05 was adopted as the criterion for statistical significance. Multiple pairwise comparisons were evaluated by applying a modified Bonferroni procedure, correcting only for comparisons with *P* ≤ 0.05 (Rom 1990). Means and standard errors (SE) are reported in text.

## Results

### Effect of weight support on muscle activity

The effect of the weight support manipulation on isometric muscle activity was confirmed in the ANOVA for background EMG (Fig. 1B, Table 1). For all muscles, there was a main effect of *support level*. There were no effects of *stimulation intensity* or *support* by *stimulation intensity* interactions. All muscles exhibited a decrease in tonic activity as the external supportive force was increased from level 1, in which no support was applied, to level 5, in which the SaeboMAS balanced the weight of the arm. Muscle activity appears to scale linearly with *support level* for AD, BB, and ECR as indicated by *R*^2^ values of 0.99, 0.99, and 0.94, respectively. For the FDI muscle, a *R*^2^ value of 0.47 likely reflects fluctuations about a resting state of muscle activity as all mean rms EMG values were ≤6 *μ*V.

**Table 1.** ANOVA of linear mixed effects models for background EMG.

Muscle	Factor	Numerator df	Denominator df	*F*	*P*
AD	Support level	4	558	223.08	**<0.0001**
	Stimulus intensity	9	558	0.15	0.9982
	Support × Stimulus	36	558	0.20	1.0000
BB	Support level	4	563	200.76	**<0.0001**
	Stimulus intensity	9	563	0.14	0.9987
	Support × Stimulus	36	563	0.13	1.0000
ECR	Support level	4	578	77.21	**<0.0001**
	Stimulus intensity	9	578	0.97	0.4658
	Support × Stimulus	36	578	0.56	0.9821
FDI	Support level	4	578	6.99	**<0.0001**
	Stimulus intensity	9	578	0.52	0.8612
	Support × Stimulus	36	578	0.48	0.9962

### Effect of weight support on motor‐evoked potentials in FDI

**Figure 2.**
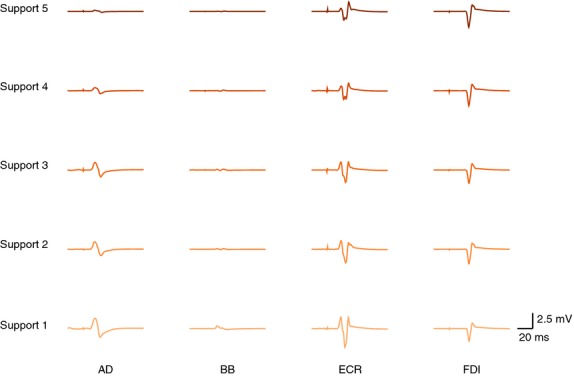
Example EMG traces showing motor‐evoked potentials (MEPs) from a single representative subject. Each trace is the average of four trials collected at an intensity 25% MSO above rest motor threshold of ECR. Support level is shown on the left. MEP areas in AD, BB, and ECR were reduced with increased weight support. For FDI, MEP area increased with increased weight support.

**Figure 3.**
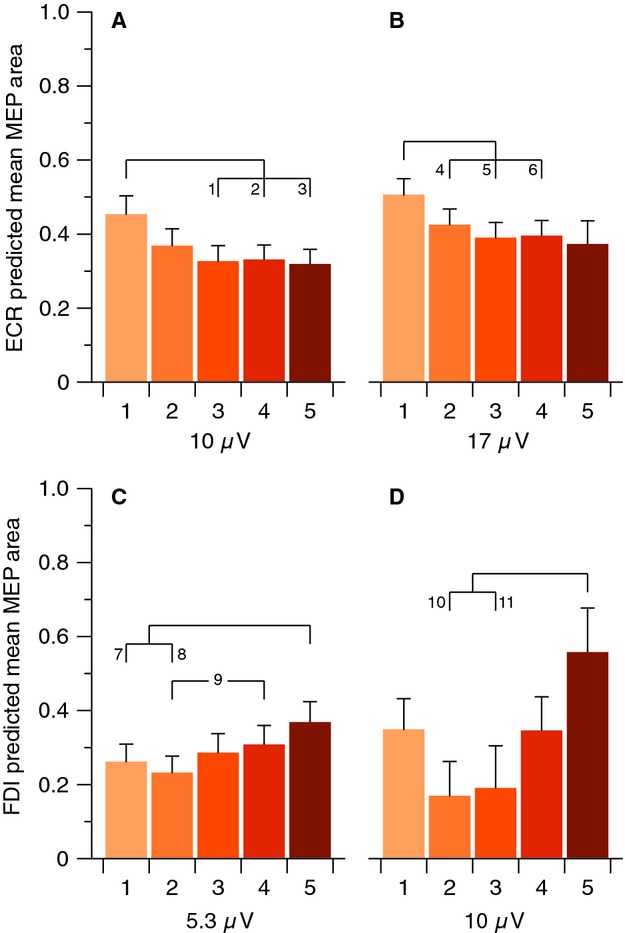
Predicted mean MEP areas for different support levels at specified values of background EMG activity. (A) For ECR at 10 *μ*V, pairwise tests showed comparisons 1 (*P* = 0.0013), 2 (*P* = 0.0011), and 3 (*P* = 0.0009) to be significant after correction. (B) For ECR at 17 *μ*V, the mean value of background EMG, pairwise tests showed comparisons 4 (*P* = 0.0121), 5 (*P* = 0.0004), and 6 (*P* = 0.0007) to be significant after correction. (C) For FDI at 5.3 *μ*V, the mean value of background EMG, pairwise tests showed comparisons 7 (*P* = 0.0006), 8 (*P* < 0.0001), and 9 (*P* = 0.0068) to be significant after correction. (D) For FDI at 10 *μ*V, pairwise tests showed comparisons 10 (*P* = 0.0041) and 11 (*P* = 0.0194) to be significant after correction. All MEP areas are normalized relative to maximum. Error bars represent standard error of the mean.

Example MEP traces are shown in Fig. 2. For the FDI muscle, the linear mixed‐effects analysis of mean MEP area revealed main effects of both *support level* and *stimulus intensity* (Table 2). There was no significant effect of *background *emg on mean MEP area, nor were there significant interaction effects. There was a trend toward an interaction between *support level* and *background* EMG. To conduct pairwise comparisons of MEP area at different support levels, the linear mixed effects model was used to predict these values at specified levels of the background EMG covariate (Fig. 3C and D). The consistent finding at both specified values of background EMG was a greater predicted mean MEP area at *support level* 5.

**Table 2.** ANOVA of linear mixed effects model for FDI MEP area.

Factor	Numerator df	Denominator df	*F*	*P*
Support level	4	528	5.36	**0.0003**
Stimulus intensity	9	528	211.62	**<0.0001**
Background EMG	1	528	0.88	0.3497
Support × Stimulus	36	528	0.83	0.7505
Support × Background	4	528	2.34	0.0542
Stimulus × Background	9	528	1.41	0.1805
Support × Stimulus × Background	36	528	1.10	0.3248

### Stimulus–response curves in FDI

**Figure 4.**
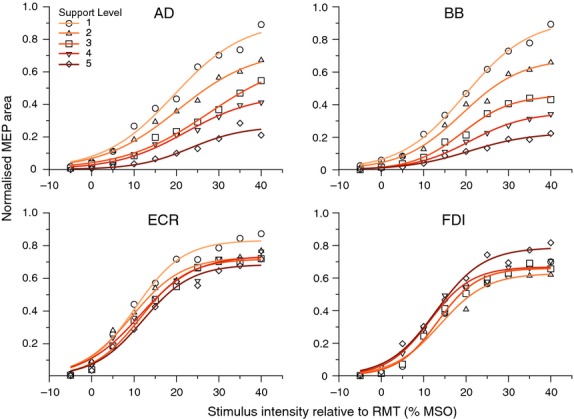
Stimulus–response regression curves fit to group mean MEP area. For ECR (bottom‐left), the plateau of the curve for support level 1 was shifted significantly upward compared to curves for support levels 3 and 5. For FDI (bottom‐right), the plateau of the curve for support level 5 was shifted significantly upward compared to curves for all other support levels. Stimulus intensities are expressed in units of stimulator output as the difference between test stimulus and RMT of ECR.

Stimulus–response curves fit to group mean MEP areas using nonlinear regression are shown in Fig. 4. The omnibus extra sum‐of‐squares F‐test indicated that individual SR curves for each *support level* fit the data better than a single global curve (*F*_(12,35)_ = 5.96, *P *<**0.0001). Follow‐up pairwise tests revealed that the curve for *support level* 5 was shifted upward compared to those for all other levels (**4**,* F*_(3,14)_ = 5.57, *P *=**0.0100 | **3**,* F*_(3,14)_ = 10.81, *P *= 0.0006 | **2**,* F*_(3,14)_ = 15.90, *P *<**0.0001 | **1**,* F*_*(*3,14)_ = 8.43, *P *=**0.0019). Furthermore, the curve for level 3 was shifted significantly upward compared to level 1 (*F*_(3,14)_ = 5.63, *P *=**0.0096). When the slope parameter was constrained to be shared among the curves for each *support level*, the extra sum‐of‐squares F test revealed no difference compared to the curves in which slope was unconstrained (*F*_(4,35)_ = 0.39, *P *=**0.8165). There was also no difference in the s50 parameter (*F*_(4,35)_ = 0.13, *P *=**0.7259). The plateau was found to be different between support levels (*F*_(4,35)_ = 6.88, *P *=**0.0003). Follow‐up comparisons revealed the plateau of level 5 (0.7878 ± 0.028) was greater than the plateaus of all other support levels (**4**, 0.6693 ± 0.019, *F*_(1,14)_ = 10.59, *P *=**0.0058 | **3**, 0.6609 ± 0.017, *F*_(1,14)_ = 13.59, *P *= 0.0024 | **2**, 0.6289 ± 0.027, *F*_(1,14)_ = 12.44, *P *=**0.0033 | **1**, 0.6631 ± 0.023, *F*_(1,14)_ = 9.83, *P *=**0.0073).

### Effect of weight support on motor‐evoked potentials in ECR

For the ECR muscle, the linear mixed‐effects analysis of mean MEP area revealed main effects of *support level, stimulus intensity*, and *background* EMG and an interaction between *stimulus intensity* and *background* EMG (Table 3). To conduct pairwise comparisons of MEP area at different *support levels*, the linear mixed effects model was used to predict these values at specified levels of the *background* EMG covariate (Fig. 3A and B). There was a similar trend at both specified values of *background* EMG. Predicted mean MEP area was greater at *support level* 1, with no differences between the higher levels of support.

**Table 3.** ANOVA of linear mixed effects model for ECR MEP area.

Factor	Numerator df	Denominator df	*F*	*P*
Support level	4	528	21.31	**<0.0001**
Stimulus intensity	9	528	244.60	**<0.0001**
Background EMG	1	528	41.24	**<0.0001**
Support × Stimulus	36	528	0.62	0.9630
Support × Background	4	528	0.78	0.5397
Stimulus × Background	9	528	2.18	**0.0223**
Support × Stimulus × Background	36	528	0.92	0.6084

### Stimulus–response curves in ECR

Following nonlinear regression (Fig. 4), the omnibus extra sum‐of‐squares F‐test indicated that individual curves for each *support level* fit the data better than a single global curve (*F*_(12,35)_ = 5.91, *P *<**0.0001). Follow‐up pairwise tests revealed that the curve for *support level ***1** was different than those for all other levels (**2**,* F*_(3,14)_ = 5.79, *P *=**0.0087 | **3**,* F*_(3,14)_ = 13.30, *P *=**0.0002 | **4**,* F*_(3,14)_ = 7.72, *P *= 0.0028 | **5**,* F*_*(*3,14)_ = 18.51, *P *< 0.0001). When the slope parameter was constrained to be shared among the curves for each *support level*, the extra sum‐of‐squares *F* test revealed no difference compared to the curves in which slope was unconstrained (*F*_(4,35)_ = 0.22, *P *=**0.9282). There was similarly no difference in the s50 parameter (*F*_(4,35)_ = 1.16, *P *=**0.3438). The plateau parameter was, however, found to be different between support levels (*F*_(4,35)_ = 4.75, *P *=**0.0036). Follow‐up comparisons revealed the plateau of level **1** (0.8332 ± 0.026) was significantly greater than the plateaus of level **3** (0.719 ± 0.019, *F*_(1,14)_ = 10.79, *P *=**0.0054) and level **5** (0.686 ± 0.026, *F*_(1,14)_ = 11.20, *P* = 0.0048).

### Effect of weight support on motor‐evoked potentials in BB

For the *Biceps Brachii*, the linear mixed‐effects analysis of mean MEP area revealed main effects of *support level, stimulus intensity*, and *background* EMG and two‐way interactions between all factors (Table 4). Stimulus–response curves fit to group means show incremental upward shifts with decreasing levels of support and associated increases in background activity (Fig. 4).

**Table 4.** ANOVA of linear mixed effects model for BB MEP area.

Factor	Numerator df	Denominator df	*F*	*P*
Support level	4	513	147.05	**<0.0001**
Stimulus intensity	9	513	190.40	**<0.0001**
Background EMG	1	513	40.76	**<0.0001**
Support × Stimulus	36	513	2.30	**<0.0001**
Support × Background	4	513	10.57	**<0.0001**
Stimulus × Background	9	513	2.51	**0.0082**
Support × Stimulus × Background	36	513	0.63	0.9557

### Effect of weight support on motor‐evoked potentials in AD

For the *Anterior Deltoid*, the linear mixed‐effects analysis of mean MEP area revealed main effects of *support level, stimulus intensity*, and *background EMG* and two‐way interactions between all factors (Table 5). Stimulus–response curves fit to group means show incremental upward shifts with decreasing levels of support and associated increases in background activity (Fig. 4).

**Table 5.** ANOVA of linear mixed effects model for AD MEP area.

Factor	Numerator df	Denominator df	*F*	*P*
Support level	4	508	105.18	**<0.0001**
Stimulus intensity	9	508	111.51	**<0.0001**
Background EMG	1	508	30.39	**<0.0001**
Support × Stimulus	36	508	2.07	**0.0004**
Support × Background	4	508	20.73	**<0.0001**
Stimulus × Background	9	508	3.64	**0.0002**
Support × Stimulus × Background	36	508	0.86	0.7079

### Effect of weight support on SICI

Short‐latency intracortical inhibition of FDI was measured at the *minimum* and *maximum* levels of support. Initial tests confirmed that background EMG (*F*_(1,38)_ = 0.32, *P *=**0.5754) and nonconditioned MEP area (*F*_(1,12)_ = 0.06, *P *=**0.8039) did not differ between the *support levels*. An analysis of MEP area revealed a main effect of *conditioning stimulus* (*F*_(1,36)_ = 29.61, *P *<**0.0001), but no effect of *support level* (*F*_(1,36)_ = 0.20, *P *=**0.6578), or *support* by *conditioning stimulus* interaction (*F*_(1,36)_ = 0.08, *P *=**0.7786). After calculating %SICI, we excluded one subject from further analysis because values less than 20% indicated the protocol was unsuccessful at eliciting SICI for this individual. At *support level ***1**, the mean amount of inhibition (70.7%) was greater than that at *support level ***5** (56.0%), whereas ΔINH was not different than zero (*t*_(11)_ = 0.89, *P* = 0.39).

Short‐latency intracortical inhibition of ECR was assessed in the same manner as for FDI. There was more background EMG activity at *support level ***1** (22.3 ± 2.4 *μ*V) than *support level ***5** (11.0 ± 2.0 *μ*V, *F*_(1,38)_ = 23.28, *P *<**0.0001). Similarly, nonconditioned MEP area was greater at *support level* 1 (0.0122 ± 0.0018 mV∙s) than *support level* 5 (0.0081 ± 0.0009 mV∙s, *F*_(1,12)_ = 10.66, *P* = 0.0068). The analysis of MEP area revealed main effects of the *conditioning stimulus* (*F*_(1,36)_ = 40.00, *P* < 0.0001), and *support level* (*F*_(1,36)_ = 16.77, *P* = 0.0002), but no *support level* by *conditioning stimulus* interaction (*F*_(1,36)_ = 1.05, *P* = 0.3113). The same subject was excluded from ECR SICI analysis because SICI was not successfully elicited. At *support level* 1, the mean amount of inhibition (48.9%) was less than that at *support level* 5 (58.5%). The direct test of change in inhibition (ΔINH) revealed a main effect of *support* on the amount of SICI (*t*_(11)_ = 2.63, *P* = 0.023). Because the direction of ΔINH differed between ECR and FDI, we compared ΔINH between the two muscles using a paired sample *t*‐test. This revealed a nonsignificant trend (*t*_(11)_ = 2.15, *P* = 0.055).

## Discussion

This is the first study to examine corticomotor excitability and M1 intracortical inhibition across the forearm and hand during systematic variation in weight support of the proximal upper limb. Consistent with our hypothesis, we observed increased corticomotor excitability (CME) in ECR at the lowest level of weight support. In contrast, CME in FDI displayed the opposite trend, being elevated at the highest level of weight support. Modulation in CME occurred independently of any differences in task requirements for these muscles. Modulation of SICI with weight support equivocally supported the CME data in both muscles. Overall, these results support a model of integrated control of the upper limb that is mediated at least in part via cortical mechanisms. These novel results may inform clinical applications of weight support such as upper limb rehabilitation after stroke (Prange et al. 2009a).

### Interactions between weight support, EMG activity, and MEP size

Weight support affected tonic muscle activity across the upper limb. Briefly, tonic activity across muscles decreased in a linear manner with weight support (Fig. 1B), with *anterior deltoid* having the greatest amplitude of activity and largest difference between low and high levels of weight support. This is consistent with its role as the prime mover for shoulder flexion and principal antigravity muscle for the posture examined (Prange et al. 2009b). Elbow flexion occurred in the horizontal plane, and therefore the effect of weight support on *biceps brachii* activity cannot be explained by mechanical task requirements. Similarly, *extensor carpi radialis* had no differential mechanical requirements and still exhibited a trend of increased tonic activity as support decreased, despite instructions to maintain relaxation. Furthermore, the differences in BB and ECR activity across support levels cannot be explained by the requirement to stabilize the arm position on target; horizontal forces did not change across conditions and successful maintenance of arm position in the horizontal plane was accomplished when muscle activity was at its minimum. The overall trend for weight support to decrease tonic activity in BB and ECR reflects a common drive across the upper limb including forearm. Conversely, FDI did not share any common drive as it remained at resting levels across all support levels.

The effect of weight support on MEP area in AD and BB indicate a progressive increase in CME above and beyond the effect of increased tonic background activity. This finding implies that excitability of motor neurons in M1 is modulated in response to weight support. This was determined by including background EMG as a covariate factor in our linear mixed effect analyses of MEP area, which showed a separate effect of support level as well as an interaction with background EMG. Thus, an upregulation of CME appears to subserve both voluntary contractions in AD and involuntary tonic activity in BB.

In ECR, increasing weight support resulted in a similar pattern of decreasing tonic activity, but a dissimilar pattern of CME changes. Although MEPs were largest in ECR at the lowest level of support as observed for AD and BB, ECR MEP areas did not decrease progressively with increased weight support (Fig. 3). It is not known why the extent of tonic activity in ECR and increase in MEP area with partial weight support dissociate. This may reflect a failure to experimentally detect a small effect, or the activity of a separate neural circuit. Nevertheless, the mechanism facilitating greater CME in ECR with low levels of support is not strictly related to tonic background activity or task requirements.

The pattern of CME modulation in FDI differed to that of the more proximal muscles with the greatest CME observed at the highest support level. The difference in MEP size across support levels is particularly interesting because FDI played no role in the task and remained at rest throughout the experiment. It is unlikely that recruitment of the FDI motoneuron pool differed substantially across support levels, as there was no effect of background EMG on MEP area. Pairwise comparisons of predicted mean MEPs corroborate the finding that CME was particularly elevated with support level 5. As was the case in ECR, the irregular change of CME across support levels implicates a threshold effect in the modulatory mechanisms. If FDI was not integrated as part of the muscle linkage in the examined task, it could be subject to surround inhibition within M1 (Beck and Hallett 2009, 2011). The increase in CME with support level 5 is consistent with a lifting of surround inhibition after activity of nearby CM neurons projecting to proximal muscles dropped below a threshold (Capaday et al. 2013). In short, the absence of substantial changes in tonic activity is indicative that FDI was not subject to a common drive with the proximal muscles (Devanne et al. 2002; Dominici et al. 2005). However, modulation of CME across support levels suggests an interaction between proximal and distal muscle representations at least, in part, via intracortical mechanisms.

### Neural mechanisms for integrated control of the upper limb

In this study, decreasing weight support reduced the amount of SICI acting on ECR representations. If this is indeed a dynamic functional linkage between ECR and proximal muscle representations in M1, its emergence only at the lowest support level is indicative of its functional role. That is, there may be a threshold of AD activity that is required to activate the intracortical networks responsible for lifting inhibition of other components like ECR, similar to that observed in the context of other studies examining proximal–distal linkage in the upper limb (Park et al. 2001; Devanne et al. 2002). For example, Devanne and colleagues also reported a facilitation of CME in ECR when AD was active in a pointing task (Devanne et al. 2002). The present findings indicate that this threshold may be similar to the activity required for an unsupported natural forward reach.

The functional linkage between muscles across the upper limb may arise from both subcortical and cortical mechanisms (Bizzi and Cheung 2013; Capaday et al. 2013). For example, common neural drive to multiple muscles may be mediated subcortically through divergence of descending corticomotor pathways (McKiernan et al. 1998), or via spinal interneuron networks (Bizzi et al. 1991). In theories which espouse modular motor control, spinal interneuron networks constrain muscle synergies by producing relatively stable patterns of activity across a subset of muscles (Lee 1984; Bizzi and Cheung 2013). At the cortical level, common neural drive may arise via dynamic linking of points within M1. For example, Schneider and colleagues demonstrated the formation of a functional linkage between remote motor areas of cat motor cortex using microstimulation that emerged with the blockade of GABA_A_ receptors from focal application of bicuculine (Schneider et al. 2002). In the hemiparetic upper limb, EMG biofeedback training can facilitate relaxation of flexors and recruitment of extensors during resting gravity‐compensated conditions. However, during unsupported reaching movements agonist–antagonist coactivation is scaled down but not eliminated by the same biofeedback (Wolf and Binder‐MacLeod 1983; Wolf et al. 1994). These previous findings are indicative that additional linking mechanisms are recruited as a function of overall muscle activity. Taken together, the effects of weight support on proximal–distal linkages are likely mediated through both cortical and subcortical mechanisms.

Reduced intracortical inhibition may also have contributed to the CME increases observed in FDI with weight support. Previous studies have concluded FDI is not part of a proximal–distal functional linkage (Devanne et al. 2002; Dominici et al. 2005). Thus, the present pattern of CME modulation suggests that FDI was subject to a nonspecific surround inhibition. Such local cortical interactions in M1 may be mediated by the topography of muscle representations. In animals, the forelimb area contains multiple representations of a given muscle that are noncontiguous and overlap those of other muscles (Donoghue et al. 1992; Schneider et al. 2001; Rathelot and Strick 2006). Similar overlapping architecture has been observed in humans (Sanes et al. 1995; Devanne et al. 2006). It is therefore possible that in the absence of a dynamic linkage, activity of an AD cortical point resulted in inhibition of a nearby FDI representation (Stinear and Byblow 2003). Although the modulation of SICI was not statistically significant, the trend in these data is consistent with a lifting of surround inhibition of FDI when activity in nearby cortical points for proximal muscles dropped below a threshold value at a high level of support.

### Potential limitations

There are limitations of this study. First, we did not make direct measurements of peripheral reflex or motoneuron excitability. Single‐pulse TMS probes the cumulative excitability of all neural elements along the corticomotor pathway. Therefore, any combination of cortical, subcortical or segmental circuits may contribute to observed differences in MEP area. Some modulation may have occurred at the spinal level. For example, the C3‐C4 propriospinal system integrates a variety of afferent information and modulates cortical output to forearm muscles (Pauvert et al. 1998; Pierrot‐Deseilligny 2002). Even though the static nature of the task precluded substantial differences in primary afferents from modulating the excitability of motoneurons, it may be the case that input from cutaneous pressure receptors varied with weight support and impacted on MN excitability directly or via propriospinal neurons (Garnett and Stephens 1981; Rossini et al. 1996; Tokimura et al. 2000).

Second, we did not test multiple arm positions or a dynamic task. Across the upper limb, the accessibility of a given muscle to recruitment depends upon static limb position (Dominici et al. 2005; Ginanneschi et al. 2005, 2006; Mogk et al. 2014). In dynamic tasks, premovement facilitation of CME across the hand and forearm is highly specific, both temporally and spatially (Rossini et al. 1988; Lemon et al. 1995). Intracortical inhibition as assessed with SICI can shape motor cortical output in a spatially and temporally specific manner during movement production (Stinear and Byblow 2003). Thus, the overall profile of CME and SICI across the upper limb might interact with weight support differentially depending on arm position and movement phase. The extent to which our results reflect a task‐specific functional linkage as opposed to a more persistent pattern of modulation remains a topic for future study.

Third, the paired‐pulse stimulation parameters were optimized for eliciting SICI in the resting ECR, and examined at only a single interstimulus interval. Different stimulation parameters may have yielded different results across muscles. Limitations withstanding, the present results provide valid observations about CME in the upper limb during weight support.

### Implications for the clinical application of weight support

Partial weight support may have relevance to upper limb rehabilitation after stroke (Prange et al. 2006). By globally reducing the amount of required activity in the weak or paretic upper limb, weight support facilitates movement repetition, which is known to promote adaptive cortical reorganization (Nudo et al. 1996). Additionally, our results indicate that partial weight support has potential to influence CME throughout the upper limb independently of its immediate mechanical effects on muscle activity. By creating unique neuromechanical control profiles, weight support may permit access to a range of motion otherwise unavailable to an individual with upper limb impairment resulting from stroke. Weight support has already shown promise as a therapeutic adjuvant after stroke (Amirabdollahian et al. 2007; Housman et al. 2009; Prange et al. 2009a; Krabben et al. 2012). A promising avenue for future research could be to determine how best to optimize weight support in order to mitigate the expression of stereotyped flexor and extensor synergy patterns common in stroke survivors with lingering upper limb impairment.

## Acknowledgments

The authors wish to thank April Ren and Jennifer Chin for their assistance with data collection. The SaeboMAS device was generously provided by Henry Hoffman at Saebo Incorporated. KR was supported by a University of Auckland Doctoral Scholarship.

## Conflict of Interest

None declared.
